# Pilot Study: Association Between Surgical Severity and Postoperative Point-of-Care C-Reactive Protein Levels in Dogs Undergoing Orthopedic Surgery

**DOI:** 10.3390/vetsci12121158

**Published:** 2025-12-04

**Authors:** Thirawat Sumalai, Supphathat Wutthiwitthayaphong, Sakchai Ruenphet, Thanikran Suwannachote

**Affiliations:** 1 Animal Biotechnology, Mahanakorn University of Technology, Bangkok 10530, Thailand; vet47ku@gmail.com (T.S.); wsupphathat@mut.ac.th (S.W.); sakchai@mut.ac.th (S.R.); 2 Samut Songkhram Animal Hospital, Samut Songkhram 75000, Thailand; 3 Immunology and Virology Department, Mahanakorn University of Technology, Bangkok 10530, Thailand; 4 Clinic for Small Domestic Animals and Radiology, Mahanakorn University of Technology, Bangkok 10530, Thailand

**Keywords:** C-reactive protein, orthopedic surgery, point-of-care quantitative immunoassay, surgical severity score, white blood cell

## Abstract

Point-of-care (POC) C-reactive protein (CRP) measurement is a valuable tool for monitoring postoperative inflammation in dogs. CRP was chosen for its high sensitivity and rapid kinetics, making it a more dynamic marker than traditional measures such as white blood cell (WBC) counts. However, its clinical utility is limited by the absence of evidence-based benchmarks that reflect surgical invasiveness. This prospective study aimed to evaluate the correlation between a standardized multi-level surgical-severity score and postoperative CRP kinetics measured by a quantitative POC immunoassay in canine orthopedic patients. Twenty-five dogs were classified by surgical severity (Level 3, *n* = 4, moderate severity; Level 4, *n* = 21, high severity). Serum CRP concentrations were measured serially over a 14-day postoperative (dpo) period. A strong positive correlation was found between surgical severity and peak postoperative CRP concentration (ρ = 0.76, *p* < 0.001). At 1 dpo, the higher-severity (Level 4) group showed a significantly greater and more prolonged CRP elevation (Mean ± SD: 67.24 ± 9.50 mg/L) than the moderate-severity (Level 3) group (Mean ± SD: 59.00 ± 22.00 mg/L). This dose–response pattern was further supported at 3 dpo, when 48% (12/25) of Level 4 dogs reached the analyzer’s upper quantification limit (70.0 mg/L), while none of the Level 3 dogs did. In contrast, postoperative WBC responses were inconsistent. These findings show that surgical severity is positively correlated with the magnitude of the POC-measured CRP response and provide preliminary severity-stratified benchmarks for interpreting postoperative inflammation in dogs.

## Simple Summary

When a dog recovers from bone surgery (such as for a knee or back injury), veterinarians need to know the difference between normal healing inflammation and a developing complication, like an infection. A quick blood test measuring C-reactive protein (CRP) is often used to check for inflammation, but there has been no clear guide on what a “normal” CRP level should be for different types of operations; for example, is the inflammation from a simple procedure the same as from a complex one? This study aimed to find out if more complex or “severe” orthopedic surgeries cause a different CRP response than simpler ones. Our results showed a clear connection: more invasive surgeries (like Tibial plateau leveling osteotomy or spinal repair) led to a significantly higher and more prolonged spike in CRP levels compared to less complex procedures. In contrast, traditional white blood cell counts were found to be less reliable for tracking this specific response. This research provides the first practical, severity-based guide for veterinarians, helping them to better interpret these common CRP test results, monitor a dog’s recovery, and identify potential problems much sooner.

## 1. Introduction

Surgical intervention is essential for many orthopedic conditions in dogs, which are a common cause of pain and debilitation. Orthopedic surgery often involves substantial soft tissue dissection, bone manipulation, and the implantation of foreign materials, all of which strongly stimulate the systemic inflammatory response (SIR) [1]. Although this response is essential for healing, excessive or dysregulated inflammation can be detrimental, contributing to increased postoperative pain, delayed wound healing, and a higher risk of surgical site infections (SSIs) [2]. The magnitude of this response is generally proportional to the extent of surgical trauma [3]. Therefore, objectively quantifying postoperative inflammation is crucial. Veterinarians must distinguish between an expected physiological recovery and a pathological complication. Failure to do so can delay intervention and compromise patient recovery.

Traditionally, postoperative monitoring relies on subjective clinical signs or conventional biomarkers such as the white blood cell (WBC) count. However, the WBC count has notable limitations; it demonstrates relatively slow kinetics and is strongly influenced by non-inflammatory factors, including stress and corticosteroid administration, making it a less specific and reliable indicator of true inflammatory burden [4,5,6]. To address these limitations, the acute phase response (APR) offers a suite of more sensitive and informative biomarkers [7,8,9]. The APR is driven by pro-inflammatory cytokines [10], among which C-reactive protein (CRP) has emerged as a key indicator of systemic inflammation in canine medicine [11,12,13]. As a positive acute phase protein, CRP is synthesized by the liver in response to cytokines—primarily interleukin-6 (IL-6) [10]. Its biological functions include opsonization and complement activation, enabling efficient targeting of pathogens and damaged cells for clearance.

Following an inflammatory stimulus, serum CRP concentrations can rise 10- to 100-fold within hours, typically peaking between 24 and 48 h [14]. Its short plasma half-life (~19 h) contributes to its highly dynamic behavior and allows it to closely reflect real-time inflammatory status [15]. A decreasing CRP concentration indicates resolution of inflammation, whereas a sustained elevation or a secondary increase strongly suggests postoperative complications [16]. This reliability has been demonstrated across a wide range of canine diseases [17,18,19,20,21]. Although CRP typically shows the most pronounced elevations in acute inflammation, it may also remain moderately increased in chronic conditions [12], supporting its role as a general indicator of inflammatory activity rather than one limited strictly to the acute response.

While postoperative CRP elevation is predictable, its clinical utility is limited by the absence of procedure-specific benchmarks. The magnitude of the CRP response is known to correlate with the degree of surgical trauma [3,22,23], yet linking CRP values to actual trauma severity requires standardized scoring systems. This gap creates uncertainty in interpretation: without established benchmarks, a high CRP value becomes difficult to assess. Clinicians cannot determine whether an elevated CRP level reflects a normal response to a highly invasive procedure or signals a complication following a less invasive one. This uncertainty constrains the diagnostic and prognostic usefulness of CRP, even though point-of-care (POC) analyzers [24,25] have made CRP testing widely accessible in routine practice [26,27]. Although POC-CRP testing can provide an early indication of postoperative complications [13,28], studies correlating multi-level surgical severity score [3,23] with POC-derived CRP kinetics in orthopedic patients remain scarce [29,30]. Establishing this relationship is essential for improving postoperative assessment and decision-making.

Therefore, the primary objective of this study was to evaluate the association between a five-level surgical severity score and the kinetics of serial postoperative CRP concentrations in dogs undergoing orthopedic procedures. A secondary aim was to provide clinicians with clearer expectations regarding the inflammatory response associated with different levels of surgical invasiveness, thereby improving the clinical value of POC-based CRP measurement in postoperative management.

We hypothesized that surgeries categorized with higher severity scores would induce a greater and more prolonged postoperative increase in serum CRP concentrations compared with those assigned lower severity scores.

## 2. Materials and Methods

### 2.1. Ethical Approval

The animal use protocol was approved by the Animal Research Ethics Committee of the Faculty of Veterinary Medicine, Mahanakorn University of Technology, Thailand (Protocol No. ACUC-MUT-2025/004).

### 2.2. Study Animals

A total of 25 client-owned dogs presenting for orthopedic surgery were prospectively enrolled in this study. The cohort included 17 males and 8 females representing 11 breeds. Details information for each dog, including gender, body weight, diagnosis, surgical technique, and assigned severity level, is provided in Table 1. The sample size (*n* = 25) reflected the clinical caseload available during the study period and was obtained through convenience sampling, and no formal a priori sample size calculation was performed.

### 2.3. Inclusion and Exclusion Criteria

Dogs were eligible for inclusion if they were client-owned, scheduled for an orthopedic procedure, and considered healthy enough to undergo general anesthesia based on a pre-surgical physical examination and routine bloodwork. In addition, written informed consent was obtained from all owners prior to enrollment. Dogs were excluded if they had evidence of concurrent systemic infectious or inflammatory disease, had received anti-inflammatory medications (e.g., corticosteroids or non-steroidal anti-inflammatory drugs) within 14 days before surgery, or had major comorbidities (e.g., neoplasia or severe organ dysfunction) that could independently influence the acute phase response.

With respect to pre-surgical analgesia, the mandatory 14-day washout period for anti-inflammatory medications meant that most dogs, particularly those with chronic orthopedic conditions such as CCLr, likely received no analgesic therapy before admission. However, in cases of acute trauma (e.g., spinal dislocations or fractures), analgesia was administered upon admission as part of the standardized anesthetic premedication protocol (see Section 2.5), immediately prior to surgery. The potential impact of this unmanaged pre-operative pain on baseline inflammatory markers is discussed in detail in the discussion section.

### 2.4. Surgical Procedures and Severity Classification

The orthopedic procedures performed in this study included hemilaminectomy, canine vertebral stabilization and rigid fixation (CVSRF), tibial plateau leveling osteotomy (TPLO), femoroacetabular head ostectomy (FHO), and others, as summarized in Table 1. To objectively quantify the degree of surgical trauma, a 5-level severity classification system was employed. Although this scale has not been formally validated in veterinary medicine, it was adapted from established principles of surgical invasiveness [3,23] to facilitate its practical application in this study. The system categorizes orthopedic surgeries according to their duration, the extent of bone manipulation involved, and the overall technical complexity (Table 2).

A qualified veterinary surgeon, blinded to the biomarker results, assigned each procedure a severity score immediately after completion. The severity score assignment was primarily guided by the specific surgical procedure performed, as listed in the ‘Example’ column. The ‘Duration’ and ‘Level of bone manipulation’ columns serve as general descriptors rather than independent criteria. Therefore, a procedure’s classification (e.g., TPLO as Level 4) was definitive, even if its specific duration slightly deviated from the listed time range.

### 2.5. Anesthetic Protocol

All dogs were managed using a standardized anesthetic protocol to minimize pharmacological variability during surgery. Premedication included butorphanol (0.2 mg/kg IV) and diazepam (0.2 mg/kg IV). Anesthesia was induced with propofol to effect (2–4 mg/kg IV). Maintenance was achieved with isoflurane (1.5–2.5%) in 100% oxygen, delivered via a cuffed endotracheal tube. Additionally, all patients received intravenous fluid therapy (Lactated Ringer’s solution) at a rate of 5 mL/kg/h throughout the procedure.

### 2.6. Postoperative Management and Clinical Assessment

Postoperative care was standardized for all patients to ensure consistent management. Prophylactic antibiotic therapy consisted of Ceftriaxone (25 mg/kg, IM, once daily) administered for 3–5 days. A standardized multimodal analgesic protocol was implemented, as follows. For the first 12–24 h, Butorphanol (0.4 mg/kg, IM) was administered every 4 h. After this period, if the animal was comfortable and eating, analgesia was transitioned to oral Tramadol (4 mg/kg, PO) every 12 h for 5–7 days.

Additional non-pharmacological measures included the application of cold packs to the surgical site for the first 3 postoperative days. Patients were monitored daily by a veterinarian blinded to the CRP results for clinical signs. Monitoring included pain assessment using the Glasgow Composite Measure Pain Scale—Short Form, evaluation of lameness, appetite, and surgical site integrity (including swelling, discharge, or dehiscence). No major postoperative complications, such as surgical site infection or implant failure, were observed in any of the 25 dogs during the 14-day study period. Standard rehabilitation, including restricted leash walks, was advised for all patients. Specific passive range of motion exercises were initiated 5–7 days postoperatively, as appropriate for each procedure.

### 2.7. Sample Collection

Venous blood samples were collected from the cephalic vein at seven predefined time points: before surgery (Day 0) and on postoperative days 1, 3, 5, 7, 10, and 14. Each sample was divided into EDTA tubes for complete blood count (CBC) and serum separator tubes for CRP determination. Serum was separated by centrifugation at 1500× *g* for 10 min and analyzed immediately for CRP concentration.

### 2.8. Inflammatory Markers

Canine C-reactive protein (CRP) concentrations were measured using an automated biochemistry analyzer (Fuji DRI-CHEM FDC NX50, FUJIFILM Europe GmbH, Düsseldorf, Germany) together with FDC vc CRP-P slides, following the manufacturer’s guidelines. This assay is based on an enzyme immunoassay (EIA) method that utilizes reflectance spectrophotometry. The established reference interval for healthy dogs using this system is <1.0 mg/dL (10 mg/L). According to the manufacturer’s protocol, CRP slides were stored below 0 °C and thawed to room temperature within their packaging prior to use. For each measurement, 10 µL of serum was mixed with a dedicated CRP sample diluent in a designated mixing cup before analysis. The analyzer provides a measurement range of 0.3–7.0 mg/dL (3–70 mg/L) for canine CRP and permits sample dilution, when necessary, with an approximate total analysis time of 5 min per sample.

Additionally, a complete blood count (CBC) was performed using an analyzer (ProCyte Dx, IDEXX Laboratories, Inc., Westbrook, ME, USA) to determine total white blood cell (WBC), neutrophil, and red blood cell (RBC) counts.

### 2.9. Statistical Analysis

All statistical analyses were performed using GraphPad Prism version 9.0 (GraphPad Software, San Diego, CA, USA). Data normality was assessed using the Shapiro–Wilk test. Although visual inspection and statistical testing indicated potential skewness in some parameters (evidenced by differences between mean and median values), repeated-measures ANOVA was retained to evaluate interaction effects across time points. To mitigate the limitations associated with small sample size and distributional assumptions, and to better assess clinical relevance beyond *p*-values alone, the analysis was supplemented with effect-size estimates. Temporal changes in CRP and total WBC concentrations across seven time points were evaluated using repeated-measures ANOVA, followed by Tukey’s post hoc test for pairwise comparisons against the preoperative baseline (0 dpo) when significant effects were detected. For comparisons between severity groups, the mean difference (MD) and 95% confidence intervals (95% Cl) were calculated to quantify the magnitude of observed effects. Data are presented descriptively as Mean ± Standard Deviation (SD), with medians included for additional clarity. Scatter plots with Mean ± SD overlays were also included to visually demonstrate the distribution and variability of the data.

Spearman’s rank correlation, a non-parametric test robust to ordinal data and unbalanced group sizes (*n* = 4 vs. *n* = 21), was first employed to assess relationships between surgical severity scores (ordinal scale) and peak postoperative CRP and WBC concentrations for each dog. The second analysis used Spearman’s correlation to evaluate relationships between mean CRP concentrations of severity level 3 and level 4 groups across all seven measurement time points. The strength of each correlation was interpreted using the corresponding rho (ρ) value.

Finally, to assess the independent effect of surgical severity while controlling for potential covariates, a multivariate general linear model (GLM) was applied. Peak postoperative CRP concentration was set as the dependent variable, surgical severity (level 3 vs. 4) as the fixed factor, and patient weight and actual surgical duration (in minutes) as covariates. For all analyses, statistical significance was set at *p* < 0.05.

## 3. Results

### 3.1. Study Population and Surgical Classification

A total of 25 client-owned dogs that underwent orthopedic surgery were included in this study. The cohort comprised various breeds, with the most common being Thai Ridgeback (*n* = 11), Alaskan Malamute (*n* = 3), and Siberian Husky (*n* = 2). The surgical procedures were classified according to a 5-level severity scale based on surgical invasiveness and technical complexity. Four dogs (16%) underwent procedures classified as severity level 3, whereas 21 dogs (84%) underwent procedures classified as severity level 4. The most frequently performed surgery was tibial plateau leveling osteotomy (TPLO) for cranial cruciate ligament rupture (*n* = 13), followed by canine vertebral screw and rod fixation (CVSRF) for spinal dislocations (*n* = 5). Demographic and surgical characteristics of all dogs are summarized in Table 1. Due to the substantial imbalance between the severity groups (Level 3, *n* = 4; Level 4, *n* = 21), the results that follow should be interpreted as exploratory.

### 3.2. Postoperative C-Reactive Protein Concentrations

**Figure 1:**
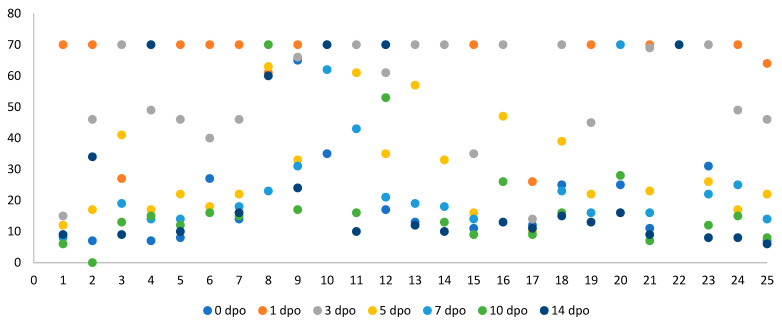
The scatter plot displays serum C-reactive protein (CRP) values (mg/L) for 25 individual dogs at seven time points: pre-operation (0 dpo) and 1, 3, 5, 7, 10, and 14 days post-operation.

The overall kinetics of serum CRP concentrations for all 25 dogs are shown in Figure 1. The preoperative (0 days post-operation, dpo) CRP level was 24.74 ± 22.12 mg/L (Median: 13.0 mg/L). Following surgery, CRP concentrations rose sharply, reaching a peak of 65.92 ± 12.05 mg/L at 1 dpo (Median: 70.0 mg/L), indicating an acute postoperative inflammatory response. A repeated-measures ANOVA confirmed a highly significant effect of time on CRP concentrations (*p* < 0.001). Post hoc analysis (Turkey’s test) confirmed that the 1, 3, and 5 dpo time points were all significantly higher than the baseline at 0 dpo (*p* < 0.05). Notably, at this time point, most dogs (18/25; 72%) reached or exceeded the analyzer’s upper quantification limit (70 mg/L).

**Figure 2:**
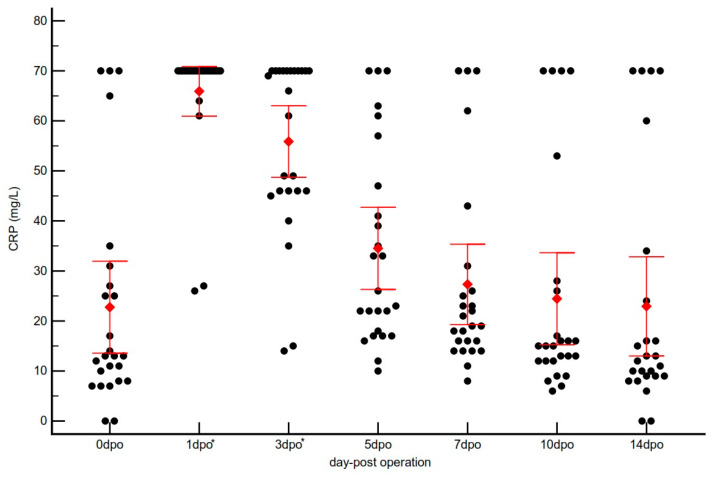
Overall postoperative kinetics of serum C-reactive protein (CRP) for all 25 dogs. The scatter plot displays individual values (black dots) and Mean ± SD (red diamond and bars) at each time point. dpo = days post-operation. * Indicates a statistically significant difference (*p* < 0.001) compared to the preoperative baseline (0 dpo), as determined by repeated-measures ANOVA with Turkey’s post hoc test.

Thereafter, CRP concentrations declined progressively, with 55.88 ± 17.33 mg/L at 3 dpo (Median: 66.0 mg/L), 34.52 ± 19.89 mg/L at 5 dpo (Median: 26.0 mg/L), 25.54 ± 17.68 mg/L at 7 dpo (Median: 19.0 mg/L), and 22.54 ± 20.60 mg/L at 10 dpo (Median: 15.0 mg/L). This pattern reflects the gradual resolution of inflammation. By 14 dpo, the CRP concentration had returned to 24.91 ± 24.03 mg/L (Median: 13.0 mg/L), closely approximating the preoperative baseline. Individual CRP profiles for all 25 dogs are presented in Figure 2.

### 3.3. C-Reactive Protein Concentrations Based on Surgical Severity Score

When stratified by surgical severity, distinct CRP response patterns were observed between the severity 3 and severity 4 groups.

**Figure 3:**
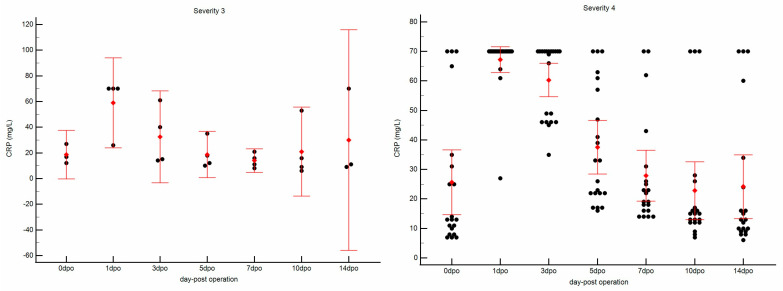
Comparison of serum C-reactive protein (CRP) concentrations between surgical severity level 3 (**left**) and level 4 (**right**) groups at each postoperative time point. The scatter plot illustrates individual data points (black dots) together with the Mean ± SD (red diamonds and bars) for each time point.

Severity 3 Group: The four dogs in this group showed a comparatively milder and shorter-lived CRP response, with a preoperative CRP of 18.67 ± 7.64 mg/L (Median: 17.0 mg/L) (Figure 3(left)). The postoperative peak at 1 dpo was 59.00 ± 22.00 mg/L (Median of 70.0 mg/L). The CRP concentration decreased more rapidly, falling to 32.50 ± 22.49 mg/L by 3 dpo (Median of. 27.5 mg/L) and returning near baseline by 5 dpo (18.75 ± 11.35 mg/L; Median 15.0 mg/L).

Severity 4 Group: The 21 dogs in this group demonstrated a robust and sustained inflammatory response, with a preoperative CRP of 25.65 ± 23.53 mg/L (Median: 13.0 mg/L) (Figure 3(right)). At 1 dpo, nearly all dogs (18/21, 86%) reached the 70.0 mg/L measurement ceiling (Mean: 67.24 ± 9.50 mg/L; censored data), reflecting a strong acute inflammatory response. The CRP remained elevated at 3 dpo (60.33 ± 12.43 mg/L; Median 70.0 mg/L) and declined steadily thereafter. Although still elevated at 5 dpo (37.52 ± 19.90 mg/L; Median 33.0 mg/L), CRP returned near baseline by 10 dpo (22.85 ± 20.93 mg/L; Median 15.0 mg/L).

The overall kinetic profile of CRP differed significantly between the two severity groups (repeated-measures ANOVA, group effect: *p* = 0.008), indicating that dogs in the severity 4 group exhibited a higher and more prolonged inflammatory response. To further characterize this difference, effect-size estimates were calculated at key postoperative time points. At the peak of inflammation (1 dpo), the mean CRP concentration in the severity 4 group (67.24 mg/L) exceeded that of the severity 3 group (59.00 mg/L), yielding a mean difference of 8.24 mg/L (95% CI: −26.1 to 42.5 mg/L). Although the confidence interval crossed zero, the estimate suggested a trend toward greater inflammation in the higher-severity group. At 3 dpo, the sustained elevation in CRP showed a mean difference of 27.83 mg/L (95% CI: −6.9 to 62.5 mg/L), again indicating a tendency toward prolonged inflammation despite the wide confidence interval. Together, these findings support the interpretation that more severe procedures are associated with a longer and more pronounced postoperative CRP response.

### 3.4. Postoperative White Blood Cell Count

**Figure 4:**
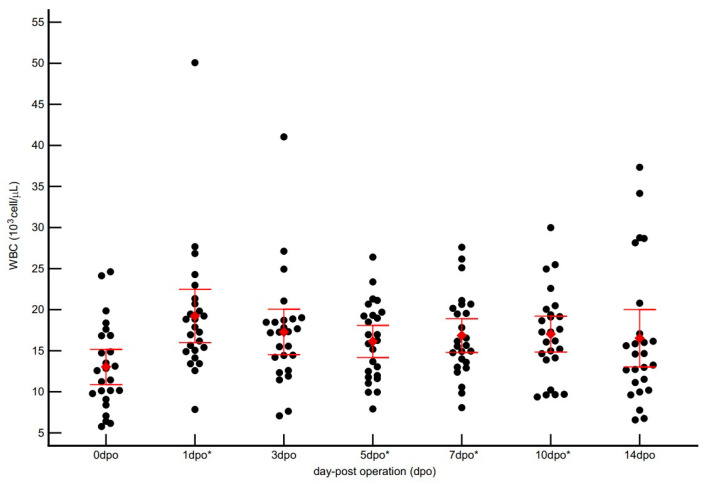
Postoperative kinetics of total white blood cell (WBC) counts (10^3^ cells/µL) in 25 dogs undergoing orthopedic surgery. The scatter dot plot shows individual WBC counts (black dots) and Mean ± SD (red diamond and bars) at each time point (dpo = days post-operation). * Indicates a significant difference (*p* < 0.05) compared to the preoperative baseline (0 dpo), as determined by repeated-measures ANOVA with Turkey’s post hoc test.

The total white blood cell (WBC) counts for all 25 dogs increased postoperatively, reflecting an acute inflammatory response (Figure 4). The preoperative WBC count was 13.1 ± 4.9 × 10^3^ cells/µL (Median: 12.58 × 10^3^ cells/µL) and peaked at 1 dpo at 19.5 ± 7.2 × 10^3^ cells/µL (Median of 17.88 × 10^3^ cells/µL), consistent with the early postoperative inflammatory surge. This temporal change was statistically significant (repeated-measures ANOVA, *p* < 0.001). Post hoc analysis. (Turkey’s test) confirmed that the 1 dpo (*p* < 0.001) and 5, 7, and 10 dpo (*p* < 0.05) time points were significantly elevated compared to baseline 0 dpo. After the peak, median WBC gradually declined but remained above baseline throughout the 14-day monitoring period, highlighting greater inter-individual variability in leukocyte dynamics following surgery.

### 3.5. Correlation and Multivariate Analysis

As hypothesized, Spearman’s rank correlation revealed a strong, statistically significant positive association between the surgical severity score (ordinal scale 3 vs. 4) and the peak postoperative CRP concentration for each dog (ρ = 0.76, *p* < 0.001). Furthermore, results from the multivariate general linear model (GLM) confirmed that surgical severity remained a significant independent predictor of peak postoperative CRP concentration (*p* = 0.005), even after controlling for the potential confounding effects of patient weight (*p* = 0.31) and actual surgical duration (*p* = 0.09).

### 3.6. Additional Hematological Parameters

**Figure 5:**
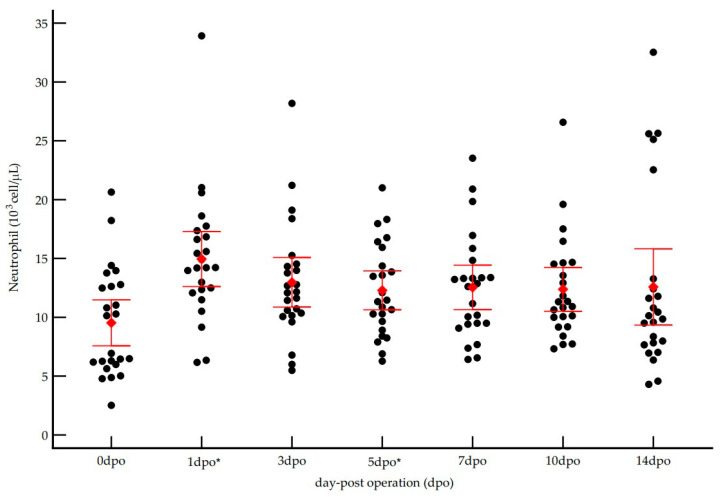
Postoperative kinetics of neutrophil counts (10^3^ cells/µL) in 25 dogs undergoing orthopedic surgery. The scatter dot plot shows individual neutrophil counts (black dots) and Mean ± SD (red diamond and bars) at each time point (dpo = days post-operation). * Indicates a significant difference (*p* < 0.05) compared to the preoperative baseline (0 dpo), as determined by repeated-measures ANOVA with Turkey’s post hoc test.

In addition to total WBC, neutrophil and red blood cell (RBC) counts were assessed. The temporal pattern of neutrophil counts closely paralleled that of total WBCs, as summarized in Table 3. Specifically, neutrophil counts increased significantly from baseline (Figure 5), peaking at 1 dpo (repeated-measures ANOVA, *p* < 0.001). In contrast, mean ± SD RBC count showed a slight, non-significant decrease across the study period (*p* > 0.05), consistent with minor intraoperative blood loss and hemodilution.

### 3.7. Postoperative Pain Assessment

Postoperative pain was monitored using the Glasgow Composite Measure Pain Scale–Short Form (GCPS-SF), as described in the Methods. Pain scores were recorded at 1, 3, 5, and 7 days postoperatively. Mean scores were highest at 1 dpo, indicating mild discomfort considered appropriate for the procedures performed. Scores then declined steadily and remained below the threshold typically requiring rescue analgesia. No major complications or episodes of unmanageable pain were reported. A summary of the pain scores is presented in Table 4.

## 4. Discussion

The key finding of this pilot study is that the postoperative CRP response, measured by a POC analyzer, correlates directly with surgical severity in dogs undergoing orthopedic procedures. Specifically, both the magnitude and duration of postoperative CRP elevation were significantly greater in the high-severity (Level 4) group compared with the moderate-severity (Level 3) group (RM-ANOVA group effect: *p* = 0.008). Furthermore, CRP was a more sensitive and dynamic biomarker of the postoperative inflammatory response than the total WBC count, which exhibited high inter-individual variability. This aligns with the established understanding that CRP demonstrates predictable, time-dependent dynamics without the marked variability observed in WBC counts. Accurate diagnosis in veterinary medicine requires interpreting multiple parameters; therefore, CRP is best interpreted as a complementary tool alongside hematological data and clinical assessment.

The kinetic profile of CRP observed in this study showed a sharp peak at 1 dpo, followed by a gradual decline. This pattern is consistent with the established understanding of the acute phase response in dogs [4,8]. CRP is synthesized in the liver, primarily under the stimulation of pro-inflammatory cytokines, most notably Interleukin-6 (IL-6), released from surgical tissue damage [31,32]. The rapid increase within hours of the inflammatory stimulus, along with its relatively short half-life, makes CRP a dynamic and sensitive marker of active inflammation [12].

The core finding of this study is the apparent differentiation in postoperative CRP response between severity 3 and severity 4 surgical groups (RM-ANOVA group effect: *p* = 0.008). The severity 4 group, which included more invasive and lengthy procedures such as TPLO and complex spinal stabilizations, showed a significantly higher and more prolonged CRP elevation compared to the severity 3 group. This dose–response relationship is biologically plausible. Greater surgical trauma, involving extensive soft tissue dissection and bone manipulation (e.g., osteotomy), leads to increased release of inflammatory mediators and, consequently, a more intense acute phase response [10,30,33,34]. This relationship was further supported by a strong positive correlation between the severity score and peak CRP levels (ρ = 0.76, *p* < 0.001). However, given the exploratory nature of this pilot study and the pronounced group imbalance, the statistical power of this correlation is limited. Thus, the reported ρ value may be unstable and should be interpreted with caution. Importantly, our multivariate analysis demonstrated that this effect was independent of patient weight or the precise surgical duration. This finding identifies the surgical classification itself as a potential key driver of the magnitude of the inflammatory response. These findings align with previous studies in both human and veterinary medicine, which have shown that the degree of surgical invasiveness correlates with the magnitude of the postoperative CRP surge [2,29,35].

In terms of clinical applicability, the study’s practical relevance is not to replace clinical judgment or other diagnostics, but to provide an objective, severity-adjusted framework for interpreting CRP values. These findings offer preliminary benchmarks for interpreting postoperative POC-CRP results. For example, a CRP value below 60 mg/L at 24 h post-operation may represent an expected response for a Level 3 procedure (e.g., FHO), whereas a value exceeding 70 mg/L is a common, uncomplicated finding for a Level 4 procedure (e.g., TPLO). This dose–response relationship is biologically plausible. The clinical risk of not recognizing this distinction is significant: a veterinarian might misinterpret a high (but normal) CRP in a Level 4 case as a complication, potentially leading to unnecessary diagnostics or antibiotic use. Conversely, a moderately high CRP in a Level 3 case might be dismissed as “expected,” thereby missing an early complication. More critically, veterinarians can use CRP kinetics as a guide. Based on this, we suggest a clinical guideline in which a peak CRP > 70 mg/L in a Level 3 case warrants immediate investigation, whereas in a Level 4 case, the trend of CRP over time is more informative. This serial monitoring, recommended for all significant orthopedic cases (Level 3 and above), can guide therapeutic decisions. Failure of CRP to decline significantly by 3–5 dpo, or any secondary rise in CRP, should prompt a thorough investigation for complications. Conversely, in a clinically improving patient, a return to near-baseline CRP may support discontinuation of NSAIDs or other analgesics.

In contrast to the rapid and pronounced response of CRP, total WBC count exhibited a distinct kinetic pattern characterized by notable inter-individual variability. Leukocytosis was observed, peaking around 1 dpo, but individual responses varied considerably. This variability underscores why CRP is often regarded as a more sensitive marker for acute systemic inflammation, although it does not replace the need for a complete blood count. WBC counts can be influenced by numerous non-inflammatory factors, including stress, excitement, and administration of corticosteroids [13,28,36,37,38,39]. These factors demonstrate why interpreting CRP kinetics alongside WBC responses provides a more comprehensive clinical picture than relying on either marker alone.

It is important to distinguish the respective roles of neutrophils and CRP. As previously noted, neutrophils constitute the primary cells of the acute inflammatory infiltrate, whereas CRP is a secondary hepatic protein. However, CRP’s utility as a systemic biomarker arises from its distinct biochemical kinetics. Neutrophil counts are influenced by multiple factors, including demargination (e.g., due to stress) and bone marrow release, which can vary considerably [36,37]. In contrast, hepatic CRP production is a highly magnified, direct response to pro-inflammatory cytokines, particularly IL-6 [10,31,32]. This results in a rapid 10- to 100-fold increase [14], making serum CRP concentration a more sensitive and less ambiguous indicator of acute systemic inflammation than neutrophil counts, which may not change as dramatically or specifically.

A key strength of this study was the standardization of anesthetic and postoperative management protocols, which allowed surgical trauma to be the primary variable influencing the CRP response. Although all anesthetic agents can have minor immunomodulatory effects, using a uniform protocol ensures that this influence is consistent across all subjects. Similarly, the standardized analgesic (butorphanol followed by tramadol) and antibiotic (ceftriaxone) regimens provided a stable baseline for assessing the inflammatory response to surgery itself. Critically, potent anti-inflammatory drugs (e.g., NSAIDs or corticosteroids) were excluded from the perioperative protocol, as stipulated by our exclusion criteria. This exclusion is essential to ensure that CRP measurements reflect true surgical inflammation. Furthermore, the absence of postoperative complications, such as infections, ensures that the measured CRP kinetics reflect an uncomplicated recovery, thereby strengthening the correlation with the initial surgical trauma.

Several limitations of the present study merit discussion. First, a primary technical limitation was the upper quantification limit of the POC analyzer (70 mg/L). At 1 dpo, most dogs in the high-severity group had CRP values exceeding this limit. This “ceiling effect” prevented accurate measurement of the true peak CRP concentration. As a result, the difference in inflammatory response between the severity 3 and 4 groups was likely underestimated. Future studies should employ analyzers with a broader dynamic range to more accurately quantify peak inflammatory responses. Second, the standardized analgesic protocol, while necessary for methodological consistency, presents limitations. The anesthetic protocol relied on butorphanol premedication and isoflurane maintenance. Butorphanol, an agonist–antagonist opioid with a relatively short duration of action, may be insufficient as the sole analgesic agent for highly invasive and lengthy (up to 3 h) orthopedic procedures. The absence of a more potent, long-acting opioid (e.g., methadone) or an intraoperative constant rate infusion (CRI) of an analgesic is a notable limitation that may have influenced perioperative stress and the subsequent inflammatory response. Third, the small and unbalanced sample size (*n* = 25) represents another limitation. In particular, the severity 3 group included only four dogs. As noted in the Methods, this convenience sample was not based on a formal power calculation. Consequently, the statistical power for comparisons was limited, reducing the generalizability of the findings. Fourth, the study population was highly heterogeneous, encompassing various breeds, surgical procedures (e.g., spinal dislocations), and chronic diseases (e.g., CCLr, hip dysplasia). This clinical diversity was reflected in the wide range of preoperative (Day 0) CRP concentrations observed (Figure 1). This baseline variability, stemming from different underlying pathologies, may have influenced the magnitude of the subsequent postoperative response and complicated the establishment of a single ‘normal’ preoperative baseline. This issue was further compounded by the mandatory 14-day anti-inflammatory drug washout period. While essential to avoid confounding the CRP results, this protocol meant that animals with painful chronic conditions or acute trauma received minimal or no analgesia prior to anesthetic premedication. This unmanaged pre-operative pain likely contributed to high variability and, in some cases, elevated baseline CRP concentrations. Furthermore, heterogeneity in surgical locations (e.g., spinal vs. articular surgeries) may involve different inflammatory pathways, although the small sample size precluded subgroup analysis. The wide preoperative CRP range also suggests that some animals may have had varying degrees of subclinical inflammation prior to surgery, potentially influencing the postoperative response. To draw a more definitive conclusion, a larger and more balanced cohort would be necessary. Nevertheless, this heterogeneity also enhances the external validity of the findings, as it reflects a typical clinical caseload presented to a veterinary hospital.

Despite these limitations, the study suggests the clinical utility of POC CRP measurement in the postoperative management of dogs. By providing an objective measure of inflammation, serial CRP monitoring can help clinicians assess whether a patient’s recovery is proceeding as expected. It can also aid. In the early detection of complications, such as surgical site infections. An unexpectedly high or secondary rise in CRP levels should prompt a thorough clinical investigation [28,40].

Future research is needed to address the limitations of this pilot study and outstanding questions. A primary goal should be to validate the 5-level severity scale and corresponding CRP benchmarks in a larger, multi-center cohort with balanced group sizes. Studies should also employ analyzers with a broader dynamic range. Further studies should correlate CRP kinetics with other inflammatory markers (e.g., pro-inflammatory cytokines, haptoglobin) and relevant clinical outcomes, such as surgical site infection or implant failure. Finally, prospective studies are needed to determine whether using these CRP guidelines for clinical decision-making (e.g., guiding analgesic duration) improves patient outcomes.

## 5. Conclusions

In conclusion, this pilot study indicates a positive correlation between surgical severity and postoperative CRP responses in dogs undergoing orthopedic surgery. The primary clinical significance of this work lies in establishing preliminary, severity-stratified CRP benchmarks. These benchmarks provide crucial context, allowing clinicians to interpret postoperative CRP values more accurately. They help differentiate expected inflammatory responses from potential complications. This supports the use of serial CRP measurements, not as a standalone test, but as a dynamic, objective tool to complement clinical assessment and guide postoperative management in dogs.

## Figures and Tables

**Table 1:** Demographic, clinical, and surgical characteristics of the 25 dogs included in the study.

No.	Breed	Gender	Weight (kg)	Diagnosis	Technique	Severity
1	Thai Ridgeback	female	15.75	Disk Herniation	Hemilaminectomy	3
2	Dachshund	male	11.1	Spinal Dislocation	CVSRF	4
3	Thai Ridgeback	male	20.4	CCLr	TPLO	4
4	Akita	male	22.5	MPL	Sulcoplasty and TTT	4
5	Thai Ridgeback	male	17.7	Spinal Dislocation	CVSRF	4
6	Border collie	female	12.00	Hip Dysplasia	FHO	3
7	Thai Ridgeback	female	23.85	CCLr	TPLO	4
8	Thai Ridgeback	male	14.8	Spinal Dislocation 2 point	CVSRF	4
9	Alaskan malamute	male	27.2	CCLr	TPLO	4
10	Thai Ridgeback	male	31.8	CCLr	TPLO	4
11	Shiba Inu	male	9.7	CCLr	TPLO	4
12	Alaskan malamute	male	22	Shoulder Luxation	Prosthetic Ligament	3
13	Pomeranian	male	7.65	CCLr	TPLO	4
14	Sibirian husky	female	32.0	CCLr	TPLO	4
15	Thai Ridgeback	female	13.4	Spinal Dislocation	CVSRF	4
16	Thai Ridgeback	female	20.0	Femoral Fracture	Plate and Screw	4
17	Labrador retriever	male	34.0	Elbow Dislocation	Prosthetic Ligament	3
18	Alaskan malamute	male	55.8	CCLr	TPLO	4
19	Thai Ridgeback	male	30.0	CCLr	TPLO	4
20	Thai Ridgeback	male	18.0	MPL	Sulcoplasty and TTT	4
21	Wales corgi	male	15.2	CCLr	TPLO	4
22	Sibirian husky	female	32.0	CCLr	TPLO	4
23	Thai Ridgeback	male	20.5	Spinal Dislocation	CVSRF	4
24	Labrador retriever	male	44.5	CCLr	TPLO	4
25	Golden retriever	female	32.0	CCLr	TPLO	4

CVSRF: Canine vertebral screw and rod fixation; CCLr: Cranial cruciate ligament rupture; TPLO: Tibial plateau leveling osteotomy; MPL: Medial patella laxation; TTT: Tibial tuberosity transposition; FHO: femoroacetabular head osteotomy.

**Table 2:** Criteria for the 5-level severity classification for veterinary orthopedic procedures used in this study.

Level	Duration	Level of Bone Manipulation	Example
1	<30 min	Minimal/None	Removal of minor implant
2	30–60 min	Drilling holes, diagnostic arthroscopy	Diagnostic arthroscopy of the stifle, pinning a phalanx
3	1–2 h	Osteotomy or simple plating	Fixation with plate and screws, Medial patellar luxation (MPL) repair with trochleoplasty
4	2–3 h	Complex and lengthy surgery	Tibial plateau leveling osteotomy (TPLO), Total hip replacement (THR)
5	>3 h	Complex osteotomy and extensive fixation	Complex pelvic or acetabular fracture repair, Revision THR

**Table 3:** Comparison of hematological parameters—total white blood cell and neutrophil counts—between baseline (0 dpo)_and days 1, 3, 5, 7, 10, and 14 postoperatively.

Time Point	Total White Blood Cells (Mean ± SD)	Neutrophils (Mean ± SD)
0 dpo	13.03 ± 5.19	9.44 ± 4.55
1 dpo	19.23 ± 7.85 *	14.82 ± 5.47 *
3 dpo	17.28 ± 6.72	12.84 ± 4.92
5 dpo	16.13 ± 4.72 *	12.11 ± 3.94 *
7 dpo	16.85 ± 4.97 *	12.45 ± 4.40
10 dpo	17.03 ± 5.29 *	12.17 ± 4.43
14 dpo	16.52 ± 8.44	12.29 ± 7.65

dpo = day-post operation * Indicates a significant difference (*p* < 0.05) compared to the preoperative baseline (0 dpo), as determined by repeated-measures ANOVA with Turkey’s post hoc test.

**Table 4:** Summary of postoperative pain scores assessed using the Glasgow Composite Measure Pain Scale—Short form (GCPS-SF), presented as mean ± SD across different time points. The GCPS-SF score ranges from 0 (no pain) to 20 (maximum pain), and scores < 5 are generally considered indicative of adequately managed postoperative pain.

Time Point	GCPS-SF Score (Mean ± SD)
1 dpo	4.8 ± 1.2
3 dpo	2.5 ± 0.9
5 dpo	1.1 ± 0.4
7 dpo	0.5 ± 0.3

dpo: day post operation.

## Data Availability

The raw data supporting the conclusions of this article will be made available by the authors on request.
